# Supplementary material for: Are midwives ready to provide quality evidence-based care after pre-service training? Curricula assessment in four countries—Benin, Malawi, Tanzania, and Uganda
Source: PLOS Glob Public Health. 2022 Sep 19;2(9):e0000605. doi: 10.1371/journal.pgph.0000605 (PMC10021168; doi:10.1371/journal.pgph.0000605)
Supplement: S2 Table — (PDF) [file pgph.0000605.s002.pdf]

S 3. Training programme, professional titles, registration, and licensing procedures

Country	Professional title	Training programme	Duration	Award	Entry requirements	Registration with the professional body	Registration and licensing to maintain practice
**Benin**	Registered Midwife	Bachelor in Midwifery	3 years	Bachelor	Baccalaureate (secondary school certificate)	National order of midwives	Authorization to work for Private Clients delivered by MoH (currently lifetime license). Authorization to work in the Public Sector is given by MoH
	Registered Nurse	Bachelor in Nursing	3 years	Bachelor	Baccalaureate (secondary school certificate)	National order of nurses	Authorization to work for Private Clients delivered by MoH (currently lifetime license). Authorization to work in the Public Sector is given by MoH
**Malawi**	Enrolled Nurse Midwife	Certificate in Nursing and Midwifery	2 Years	Certificate	Students need to have Malawi School Certificate of Education or its equivalent with six credits in Mathematics, Biology, Physical Science, English or English Language and any other two subjects	License is provided after passing licensure exams	All licenses are renewed yearly through Malawi Nurses and Midwifery Council
	Nurse- Midwife Technician	Diploma in Nursing and Midwifery	3 years	Diploma	Students need to have a full Malawi School Certificate of Education with credit passes in three Science Subjects with Biology and Physical Science being mandatory credits	License is provided after passing licensure exams	All licenses are renewed yearly through Malawi Nurses and Midwifery Council
	Registered Nurse-Midwife	Diploma in Nursing and Certificate in Midwifery	4 years	3 years Diploma in Nursing and 1 year Certificate in Midwifery	Students need to have a full Malawi School Certificate of Education with a minimum of 6 Credits. Including English and three Science Subjects with Biology and Physical Science being mandatory credits	License is provided after passing licensure exams	All licenses are renewed yearly through Malawi Nurses and Midwifery Council

Country	Professional title	Training programme	Duration	Award	Entry requirements	Registration with the professional body	Registration and licensing to maintain practice
	Registered Midwife	Bachelor in Midwifery	4 years	Bachelor	Students need to have a full Malawi School Certificate with a minimum of 6 Credits. Including English and three of Science Subjects with Biology and Physical Science being mandatory credits	License is provided after passing licensure exams	All licenses are renewed yearly through Malawi Nurses and Midwifery Council
	Registered Nurse-Midwife	Bachelor in Nursing and Midwifery	4 years	Bachelor	MSCE/O-Level credits in Biology, Physical Science, English and Mathematics. University diploma in Nursing. Be a Registered Nurse/ Midwife	License is provided after passing licensure exams	All licenses are renewed yearly through Malawi Nurses and Midwifery Council
	Advanced Midwife Practitioner	Masters of Science in Midwifery	2 years	Master	Bachelor in Midwifery	License is provided after passing licensure exams	All licenses are renewed yearly through Malawi Nurses and Midwifery Council
**Tanzania**	Enrolled Nurse- Midwife	Certificate in Nursing and Midwifery	2 years	Certificate	Certificate of Ordinary level secondary school	Tanzania Nurses and Midwives Council (TNMC)	License renewal after every 3 years and after earning some continuing professional development (CPD) points
	Registered Nurse-Midwife	Diploma in Nursing and Midwifery	3 years (and some have been trained for 4 years	Diploma	Certificate of Ordinary level secondary school	Tanzania Nurses and Midwives Council (TNMC)	License renewal after every 3 years and after earning some CPD points
	Registered Midwife	Bachelor of Science in Midwifery	4 years (for direct entrants and 3 years for in-service [previous in 2010 to 2016] and 4 years for entrants with a diploma in nursing and midwifery [currently])	Bachelor	Certificate of Advanced level secondary school [for direct entrants] and a Diploma certificate with a GPA of 3.5 or B grade [for in-service entrants with a diploma in nursing and midwifery]	Tanzania Nurses and Midwives Council (TNMC)	License renewal after every 3 years and after earning some CPD points

Country	Professional title	Training programme	Duration	Award	Entry requirements	Registration with the professional body	Registration and licensing to maintain practice
	Registered Nurse	Bachelor of Science in Nursing	4 years (for direct entrants)	Bachelor	Certificate of Advanced level secondary school (for direct entrants)	Tanzania Nurses and Midwives Council (TNMC)	License renewal after every 3 years and after earning some CPD points
	Master of Science in Midwifery and Women's Health	Master of Science in Midwifery and Women's Health	2 Years	Master	Applicants with BSc Midwifery degree or BSc Nursing degree with a GPA of at least 2.7 or a BSc Midwifery degree or BSc Nursing degree from a recognized University with a GPA of at least 3.0 will be considered if they satisfy all the general requirements set out under the regulations for a Master's degree	Tanzania Nurses and Midwives Council (TNMC)	License renewal after every 3 years and after earning some CPD points
**Uganda**	Enrolled Midwife	Certificate in midwifery	2.5 years	Certificate	Uganda Certificate of Education	Uganda Nurses and Midwives Council (UMNC)	Renewal of license is every 3 years
	Registered Midwife	Diploma in Midwifery (Upgrading, Top up)	18 months	Diploma	Certificate in Midwifery, Diploma in Nursing	UNMC	Renewal of license is every 3 years
	Registered Midwife	Diploma in Midwifery	3 years	Diploma	Uganda Advanced Certificate of Education	UNMC	Renewal of license is every 3 years
	Bachelor of Science in Midwifery	Bachelor of Science in Midwifery	4 years	Bachelor	Advanced Certificate of Education, Diploma in any health-related field	UNMC	Renewal of license is every 3 years
	Registered Master of Nursing	Nursing (Midwifery and Women's Health)	2 years	Master	Bachelor of Science in Nursing, Bachelor of Nursing Science, Bachelor of Midwifery, Bachelor of Midwifery Science	UNMC	Renewal of license is every 3 years
