# Supplementary material for: Are midwives ready to provide quality evidence-based care after pre-service training? Curricula assessment in four countries—Benin, Malawi, Tanzania, and Uganda
Source: PLOS Glob Public Health. 2022 Sep 19;2(9):e0000605. doi: 10.1371/journal.pgph.0000605 (PMC10021168; doi:10.1371/journal.pgph.0000605)
Supplement: S3 Table — (PDF) [file pgph.0000605.s003.pdf]

S4. Number of indicators in country curriculum (N) mapped to the ICM framework (%) and years of training programmes

Competency title		No. of indicators per competency in the ICM framework	Number of indicators in country curricula (N) mapped to the ICM framework (%) and years of training programmes																			
			Benin License in Midwifery (3 years)		Malawi BSc Nursing and Midwifery (4 years)		Malawi Diploma Nursing and Midwifery Technician (3 years)		Tanzania Diploma Nursing and Midwifery (3 years)		Tanzania Technician Certificate in Nursing and Midwifery (2 years)		Tanzania Revised Competency-based Curriculum for the Bachelor of Science in Nursing Programme (4 years)		Tanzania Competency - based Curriculum for Bachelor of Science in Midwifery (4 years)		Uganda BSc (upgrading) Midwifery (2.5 years)		Uganda BSc Midwifery (4 years)		Uganda Diploma Midwifery (3 years)	
		N	N	%	N	%	N	%	N	%	N	%	N	%	N	%	N	%	N	%	N	%
1. GENERAL COMPETENCIES																						
1.a Assume responsibility for own decisions and actions as an autonomous practitioner	Knowledge	5	2	40	3	60	1	20	2	40	1	20	3	60	3	60	4	80	1	20	2	40
	Skills and behaviours	4	2	50	1	25	1	25	2	50	1	25	3	75	3	75	4	100	2	50	2	50
1.b Assume responsibility for self-care and self-development as a midwife	Knowledge	1	1	100	1	100	1	100	1	100	1	100	1	100	1	100	1	100	1	100	1	100
	Skills and behaviours	6	5	83	3	50	3	50	1	17	1	17	4	67	5	83	5	83	3	50	4	67
1.c Appropriately delegate aspects of care and provide supervision	Knowledge	3	3	100	2	67	3	100	2	67	0	0	0	0	1	33	0	0	3	100	3	100
	Skills and behaviours	2	0	0	2	100	0	0	1	50	0	0	0	0	0	0	0	0	1	50	2	100
1.d Use research to inform practice	Knowledge	3	2	67	2	67	3	100	3	100	2	67	2	67	2	67	3	100	2	67	1	33
	Skills and behaviours	2	1	50	2	100	0	0	1	50	0	0	1	50	2	100	1	50	1	50	2	100
1.e Uphold fundamental human rights of individuals when providing midwifery care	Knowledge	4	0	0	4	100	1	25	2	50	2	50	2	50	3	75	3	75	3	75	1	25
	Skills and behaviours	5	1	20	1	20	1	20	1	20	0	0	1	20	3	60	0	0	4	80	0	0
1.f Adhere to jurisdictional laws, regulatory requirements, and codes of conduct for midwifery practice	Knowledge	4	3	75	2	50	4	100	3	75	3	75	4	100	4	100	3	75	2	50	3	75
	Skills and behaviours	7	2	29	7	100	5	71	4	57	4	57	4	57	6	86	6	86	5	71	3	43
1.g Facilitate women to make individual choices about care	Knowledge	3	1	33	2	67	2	67	2	67	2	67	1	33	2	67	3	100	2	67	1	33
	Skills and behaviours	4	0	0	0	0	1	25	0	0	0	0	1	25	1	25	2	50	0	0	0	0
1.h Demonstrate effective interpersonal communication with women and families, health care teams, and community groups	Knowledge	5	4	80	5	100	5	100	5	100	5	100	4	80	5	100	5	100	5	100	3	60
	Skills and behaviours	10	2	20	7	70	7	70	7	70	7	70	9	90	7	70	7	70	5	50	10	100
1.i Facilitate normal birth processes in institutional and community settings, including women's homes	Knowledge	5	2	40	3	60	3	60	3	60	2	40	2	40	2	40	4	80	2	40	0	0
	Skills and behaviours	3	0	0	1	33	0	0	0	0	0	0	2	67	2	67	2	67	1	33	0	0
1.j Assess the health status, screen for health risks, and promote general health and well-being of women and infants	Knowledge	3	3	100	3	100	3	100	3	100	3	100	2	67	3	100	3	100	3	100	3	100
	Skills and behaviours	6	2	33	4	67	3	50	3	50	3	50	4	67	6	100	5	83	5	83	3	50
1.k Prevent and treat common health problems related to reproduction and early life	Knowledge	4	4	100	4	100	4	100	4	100	4	100	4	100	4	100	4	100	4	100	4	100
	Skills and behaviours	6	4	67	4	67	4	67	4	67	4	67	3	50	4	67	5	83	5	83	4	67
1.l Recognise abnormalities and complications and institute appropriate treatment and referral	Knowledge	5	5	100	4	80	5	100	5	100	5	100	5	100	5	100	4	80	4	80	4	80
	Skills and behaviours	7	5	71	5	71	5	71	6	86	6	86	5	71	6	86	7	100	5	71	6	86
1.m Care for women who experience physical and sexual violence and abuse	Knowledge	3	0	0	3	100	0	0	3	100	0	0	2	67	0	0	0	0	1	33	1	33
	Skills and behaviours	6	0	0	5	83	1	17	4	67	1	17	1	17	1	17	2	33	2	33	2	33
1. Sub-total	Knowledge	48	30	63	38	79	35	73	36	75	30	63	32	67	35	73	37	77	33	69	27	56
	Skills and behaviours	68	24	35	42	62	31	46	34	50	27	40	38	56	46	68	46	68	39	57	38	56
**1. Total**		116	54	47	80	69	66	57	70	60	57	49	70	60	81	70	83	72	72	62	65	56
2. COMPETENCIES SPECIFIC TO PRE- PREGNANCY AND ANTENATAL CARE																						
2.a Provide pre-pregnancy care	Knowledge	3	2	67	2	67	2	67	2	67	2	67	3	100	3	100	3	100	3	100	2	67
	Skills and behaviours	4	3	75	3	75	2	50	3	75	3	75	1	25	3	75	4	100	3	75	3	75
2.b Determine health status of woman	Knowledge	4	3	75	4	100	3	75	2	50	2	50	4	100	4	100	4	100	4	100	4	100
	Skills and behaviours	7	5	71	6	86	5	71	6	86	6	86	2	29	6	86	5	71	4	57	5	71
2.c Assess fetal well-being	Knowledge	2	1	50	1	50	1	50	1	50	1	50	1	50	1	50	1	50	0	0	1	50
	Skills and behaviours	3	3	100	3	100	0	0	2	67	2	67	2	67	2	67	3	100	2	67	2	67
2.d Monitor the progression of pregnancy	Knowledge	4	2	50	4	100	2	50	3	75	3	75	4	100	4	100	4	100	2	50	3	75
	Skills and behaviours	5	3	60	4	80	3	60	4	80	4	80	3	60	5	100	4	80	3	60	3	60
2.e Promote and support health behaviours that improve wellbeing	Knowledge	7	2	29	2	29	1	14	4	57	4	57	3	43	4	57	5	71	3	43	3	43
	Skills and behaviours	5	1	20	1	20	1	20	2	40	2	40	2	40	4	80	5	100	0	0	1	20
2.f Provide anticipatory guidance related to pregnancy, birth, breastfeeding, parenthood, and change in the family	Knowledge	3	1	33	2	67	2	67	2	67	2	67	2	67	2	67	2	67	1	33	1	33
	Skills and behaviours	5	3	60	3	60	4	80	3	60	3	60	3	60	3	60	4	80	2	40	4	80
2.g Detect, stabilse, manage, and refer women with complicated pregnancies	Knowledge	4	0	0	4	100	4	100	4	100	4	100	2	50	4	100	4	100	4	100	3	75
	Skills and behaviours	5	4	80	4	80	4	80	4	80	4	80	4	80	4	80	4	80	4	80	4	80
2.h Assist the woman and her family to plan for an appropriate place of birth	Knowledge	3	1	33	1	33	2	67	2	67	2	67	2	67	2	67	1	33	0	0	0	0
	Skills and behaviours	3	0	0	1	33	1	33	1	33	1	33	2	67	2	67	2	67	0	0	1	33
2.i Provide care to women with unintended or mistimed pregnancy	Knowledge	7	0	0	0	0	0	0	1	14	1	14	0	0	0	0	4	57	3	43	2	29
	Skills and behaviours	8	1	13	2	25	1	13	1	13	1	13	0	0	1	13	2	25	3	38	3	38
ADDITIONAL SKILL*	Skills	2	0	0	0	0	0	0	0	0	0	0	0	0	0	0	0	0	0	0	0	0
2. Sub-total	Knowledge	37	12	32	20	54	17	46	21	57	21	57	21	57	24	65	28	76	20	54	19	51
	Skills and behaviours	47	23	49	27	57	21	45	26	55	26	55	19	40	30	64	33	70	21	45	26	55
**2. Total**		84	35	42	47	56	38	45	47	56	47	56	40	48	54	64	61	73	41	49	45	54
3. COMPETENCIES SPECIFIC TO CARE DURING LABOUR AND BIRTH																						
3.a Promote physiologic labour and birth	Knowledge	6	5	83	5	83	5	83	5	83	5	83	5	83	6	100	5	83	5	83	4	67
	Skills and behaviours	14	7	50	11	79	7	50	10	71	8	57	8	57	11	79	11	79	8	57	6	43
3.b Manage a safe spontaneous vaginal birth; prevent, detect and stabilise complications	Knowledge	6	5	83	6	100	6	100	6	100	6	100	4	67	6	100	6	100	6	100	6	100
	Skills and behaviours	14	9	64	9	64	10	71	11	79	10	71	4	29	10	71	13	93	8	57	10	71
3.c Provide care of the newborn immediately after birth	Knowledge	7	4	57	6	86	7	100	7	100	6	86	5	71	6	86	7	100	0	0	7	100
	Skills and behaviours	7	2	29	5	71	6	86	4	57	4	57	4	57	6	86	7	100	1	14	5	71
3. Sub-total	Knowledge	19	14	74	17	89	18	95	18	95	17	89	14	74	18	95	18	95	11	58	17	89
	Skills and behaviours	35	18	51	25	71	23	66	25	71	22	63	16	46	27	77	31	89	17	49	21	60
**3. Total**		54	32	59	42	78	41	76	43	80	39	72	30	56	45	83	49	91	28	52	38	70
4. COMPETENCIES SPECIFIC TO THE ONGOING CARE OF WOMEN AND NEWBORNS																						
4.a Provide postnatal care for the healthy woman	Knowledge	4	3	75	4	100	2	50	3	75	2	50	4	100	3	75	3	75	1	25	1	25
	Skills and behaviours	6	3	50	5	83	2	33	5	83	4	67	2	33	3	50	5	83	2	33	2	33
4.b Provide care to healthy newborn infant	Knowledge	5	2	40	3	60	3	60	4	80	3	60	2	40	2	40	4	80	4	80	4	80
	Skills and behaviours	4	1	25	3	75	3	75	4	100	2	50	4	100	3	75	4	100	4	100	3	75
4.c Promote and support breastfeeding	Knowledge	6	2	33	4	67	3	50	0	0	0	0	2	33	3	50	2	33	4	67	2	33
	Skills and behaviours	7	0	0	6	86	3	43	1	14	1	14	2	29	4	57	5	71	2	29	3	43
4.d Detect, treat, and stabilise postnatal complications in woman and refer as necessary	Knowledge	3	1	33	3	100	2	67	2	67	2	67	1	33	3	100	2	67	3	100	2	67
	Skills and behaviours	6	4	67	5	83	4	67	4	67	4	67	3	50	5	83	5	83	4	67	4	67
4.e Detect stabilise, and manage health problems in newborn infant and refer if necessary	Knowledge	5	2	40	4	80	4	80	4	80	2	40	3	60	4	80	5	100	3	60	4	80
	Skills and behaviours	6	2	33	6	100	5	83	6	100	3	50	2	33	6	100	5	83	3	50	4	67
4.f Provide family planning services	Knowledge	5	4	80	4	80	3	60	3	60	3	60	4	80	3	60	3	60	5	100	3	60
	Skills and behaviours	6	3	50	4	67	4	67	3	50	3	50	4	67	3	50	6	100	4	67	4	67
4. Sub-total	Knowledge	28	14	50	22	79	17	61	16	57	12	43	16	57	18	64	19	68	20	71	16	57
	Skills and behaviours	35	13	37	29	83	21	60	23	66	17	49	17	49	24	69	30	86	19	54	20	57
**4. Total**		63	27	43	51	81	38	60	39	62	29	46	33	52	42	67	49	78	39	62	36	57
**Total knowledge**	Knowledge	132	70	53	97	73	87	66	91	69	80	61	83	63	95	72	102	77	84	64	79	60
**Total skills and behaviours**	Skills and behaviours	185	78	42	123	66	96	52	108	58	92	50	90	49	127	69	140	76	96	52	105	57
**TOTAL all indicators**		**317**	**148**	**47**	**220**	**69**	**183**	**58**	**199**	**63**	**172**	**54**	**173**	**55**	**222**	**70**	**242**	**76**	**180**	**57**	**184**	**58**

*Prescribe, dispense, furnish or administer drugs according to scope of practice and protocol (however authorized to do so in the jurisdiction of practice) in dosages appropriate to induce medication abortion.

Perform manual vacuum aspiration of the uterus up to 12 completed weeks of pregnancy.

An additional skill is performed by midwives under either of two circumstances:

a) Midwives who elect to engage in a broader scope of practice and/or b) Midwives who have to implement certain skills to make a difference in maternal or neonatal outcome.
