# Supplementary material for: Are midwives ready to provide quality evidence-based care after pre-service training? Curricula assessment in four countries—Benin, Malawi, Tanzania, and Uganda
Source: PLOS Glob Public Health. 2022 Sep 19;2(9):e0000605. doi: 10.1371/journal.pgph.0000605 (PMC10021168; doi:10.1371/journal.pgph.0000605)
Supplement: S1 Text — (PDF) [file pgph.0000605.s004.pdf]

ALERT – Mapping of curricula

Interview questions for ALERT project lead midwives

Professional title/level of education

1. What are the professional titles of those who are recognised as skilled providers of antenatal, intrapartum, and postnatal care in your country?
2. Please list all the levels of qualification that exist for each professional title, and how this is reflected in the job title (e.g., an enrolled midwife may undertake a certificate in midwifery, but a registered midwife may undertake a diploma in midwifery).

Registration/licensing procedures

1. For each professional title, please describe their governing body (e.g., for midwives this may be the Nurses and Midwives Council).
2. For each professional title, please provide describe of how an individual who has completed their pre-service training registers with the governing body/obtains a license to practice. Please also provide information on how this registration/license to practice is maintained (e.g., individual must pay annual fees/provide evidence of continuous professional development/provide evidence of work in the field).

Professional organisations

1. Please list the different professional organisations that represent each profession in your country.
2. Please give a brief overview of the role each professional organization plays in supporting its members.
