# Supplementary material for: Are midwives ready to provide quality evidence-based care after pre-service training? Curricula assessment in four countries—Benin, Malawi, Tanzania, and Uganda
Source: PLOS Glob Public Health. 2022 Sep 19;2(9):e0000605. doi: 10.1371/journal.pgph.0000605 (PMC10021168; doi:10.1371/journal.pgph.0000605)
Supplement: S1 Table — (PDF) [file pgph.0000605.s001.pdf]

S2. Data collection form: Mapping to ICM Essential Competencies for Midwifery Practice

Country				Benin	Malawi	Malawi	Tanzania level 6	Tanzania level 5	Tanzania	Tanzania	Uganda	Uganda	Uganda
Name of curricula				Maquette Pédagogique de l'Offre de Formation de Licence Professionnelle en Sciences Infirmières et Obstétricales Option Sage-Femme	BSc in Nursing and Midwifery	Nursing and Midwifery Technicians (NMTs)	Ordinary Diploma Nursing and Midwifery	Technicain Certificate in Nuring and Midwifery	Revised Competency-based Curriculum for the Bachelor of Science in Nursing (BSCN) Programme	Competency - Based Curriculum for Bachelor of Science in Midwifery	BSc of science in midwifery (upgrading) programme	BSs midwifery	Diploma in midwifery
Publication date				2018	2019	2020	2017	2017	2020	2018	2014	2015	2015
Professional Title				Midwife	BSc in Nursing and Midwifery	Nursing and Midwifery Technician	Reg. Nurse/Midwife	Technicain Certificate in Nuring and Midwifery	Bachelor of Science in Midwifery in Nursing	Bachelor of Science in Midwifery	BSc of science in midwifery	BSs midwifery	Diploma in midwifery
Academic qualification				Prof. Licence	Bachelor	Diploma	Diploma	Certificate	Bachelor	Bachelor	Bachelor	Bachelor	Diploma
Length of course				3 years	4 years	3 years	3 years	2 years	4 years	4 years	2.5 years	4 years	3 years
Hours total				4500	Does not add up	5430	4199	2839	5264	5713	2304	5565	4170
Entry requirements				Hold a Baccalaureate C or D or any other diploma recognized as equivalent and dating from less than 2 years at the time of registration.	a) MSCE or its equivalent with 6 credits in the following subjects<br><br>1. English, Physical Science, Biology, Mathematics and any other two subjects<br>OR<br>2. English, Biology, Chemistry, Mathematics and any other two subjects<br><br>b) Candidates should be 16 or above by the date of application.	a) Has reached the age of sixteen (16) years and does not exceed the age of forty-five (45) years.<br><br>b) Provides evidence of having passed the full Malawi School Certificate of Education (MSCE) or its equivalent with six credits that include English and at least three (3) credits in science subjects, (i.e. credits in either Physical Science and/or Physics and Chemistry, Biology and any other science subject are required) plus any 2 other subjects.	The course is open to candidate who has a Certificate of Secondary Education Examination (CSEE) with minimum grade of "C" in Biology and Chemistry, pass at grade "D" in Physics/ Engineering Science and English language, pass in Mathematics is an added advantage.	The course is open to a candidate who has successfully completed NTA level 4.<br><br>NTA Level 4 requirements:The course is open to candidate who has a Certificate of Secondary Education Examination (CSEE) with minimum grade of "C" in Biology and Chemistry, pass at grade "D" in Physics/ Engineering Science and English language, pass in Mathematics is an added advantage.	Direct Entrants: Three principal passes in Physics, Chemistry and Biology at Advanced ("A") level with minimum entry point of 6 points. Whereby one must have at least C grade in Chemistry and Biology and at least D grade in Physics.<br><br>Equivalent Qualification: Holder of Diploma or Advanced Diploma in Nursing with a minimum GPA of 3.5 or B grade accredited by a relevant authority and approved by the University Senate.<br><br>The candidate should have a certificate of secondary education examination (CSEE) with three passes in Biology, Chemistry and Physics. Principal passes at any grade in Physics, Chemistry and Biology at "A" level will be an added advantage.	A holder of Diploma or Advanced Diploma in Midwifery/Nursing with a minimum GPA of 3.5 or B grade accredited by a relevant authority.<br><br>Should have a certificate of secondary education examination (CSEE) with three passes in subjects that include Biology, Chemistry, Physics, and English.<br><br>Principal passes at any grade in Physics, Chemistry, and Biology at Advanced Certificate of Secondary Examination (ACSE) will be an added advantage.<br><br>Should be registered and licensed by the Tanzania Nursing and Midwifery Council with a valid license to practice.	Uganda Registered Midwife (URM) aggregate or Diploma Comprehensive Nurse (DCN).<br><br>Currently registered with the Uganda Nurses and Midwives Council.<br><br>At least 2 years working experience, preferably in MCH related setting.	A candidate must have at least the following: A Diploma in Nursing or Midwifery or<br>A Diploma in Education majoring in Biology and Chemistry, preference being given to women applicants.<br>A Diploma in Comprehensive Nursing or<br>A Diploma in any other relevant health discipline<br>Students to be admitted under this scheme will constitute 20%.<br><br>Direct Entry Scheme<br>A candidate must have the following:<br>Uganda Certification of Education (UCE) or its equivalent with at least<br>minimum of credit passes in Mathematics and English in the same sitting.<br>Uganda Advanced Certificate of Education (UACE) (A level) or its equivalent with at least 2 principal passes in the Core Subjects Biology, Physics and Chemistry taken at the same sitting.Other relevant subjects are Geography, Agriculture and English.<br>All candidates for this programme should obtain minimum weighted points as shall be determined by the Admissions Board.	Direct entry:<br>Advanced level certificate, 1 principle pass in Biology, and 2 other subsidiary passes in any other subject, but including Chemistry.<br>Direct entry should be restricted to not more than three years after secondary school.<br>Pass a pre-entry Ministry of Education, Science, Technology and Sports oral interview.
				Included<br>Yes=x<br>No= no	Included<br>Yes=x<br>No= no	Included<br>Yes=x<br>No= no	Included<br>Yes=x<br>No= no	Included<br>Yes=x<br>No= no	Included<br>Yes=x<br>No= no	Included<br>Yes=x<br>No= no	Included<br>Yes=x<br>No= no	Included<br>Yes=x<br>No= no	Included<br>Yes=x<br>No= no
Category 1: General competencies													
1a: Assume responsibility for own decisions and actions as an autonomous practitioner													
Knowledge	Principles of accountability and transparency			no	x	x	no	no	x	no	x	x	x
	Principles and concepts of autonomy			x	x	no	x	x	x	x	x	no	no
	Principles of self-assessment and reflective practice			no	se note	no	no	no	x	x	x	no	no
	Personal beliefs and their influence on practice			no	no	no	no	no	no	no	no	no	no
	Knowledge of evidence-based practices			x	x	no	x	no	x	x	x	no	x
Skills and Behaviours	Demonstrate behaviour that upholds the public trust in the profession			no	no	no	no	no	x	x	x	no	no
	Participate in self-evaluation, peer review and other quality improvement activities			x	no	no	x	no	x	x	x	x	x
	Balance the responsibility of the midwife to provide best care with the autonomy of the woman to make her own decisions			no	no	no	no	no	no	no	x	no	no
	Explain the midwife's role in providing care that is based on relevant laws, ethics and evidence.			x	x	x	x	x	x	x	x	x	x
1b: Assume responsibility for self-care and self-development as a midwife													
Knowledge	Strategies for managing personal safety particularly within the facility or community setting			x	x	x	x	x	x	x	x	x	x
Skills and behaviours	Display skills in management of self in relation to time management, uncertainty, change and coping with stress			x	no	no	no	no	x	x	x	x	no
	Assume responsibility for personal safety in various practice settings			x	x	x	x	x	x	x	x	x	x
	Maintain up-to-date skills and knowledge concerning protocols, guidelines and safe practice			x	x	x	no	[illegible]	x	x	x	no	no
	Remain current in practice by participating in continuing professional education (for example, participating in learning opportunities that apply evidence to practice to improve care such as mortality reviews or policy reviews)			no	no	no	no	[illegible]	no	x	x	x	x

[illegible]

	Conduct a focused physical exam to assess breast changes and involution. Monitor blood loss and other body functions	x	x	x	x	x	x	x	x	x	x
	Assess mood and feeling about motherhood and demands of infant care	no	x	no	x	x	no	x	x	no	no
	Provide pain control strategies if needed for uterine contractions, and perineal trauma	no	x	no	x	no	no	no	no	no	no
	Provide information about self-care that enables mother to meet needs of newborn, e.g. adequate food, nutritional supplements, usual activities, rest periods, and household help	no	no	no	x	x	x	x	x	no	no
	Provide information about safe sex, family planning methods appropriate for the immediate postnatal period, and pregnancy spacing	x	x	no	x	x	no	no	x	x	x
**4b: Provide care to healthy newborn infant**											
Knowledge	Appearance and behaviour of infant in early life; cardio-respiratory changes related to adapting to extra-uterine life	x	x	x	x	x	x	x	x	x	x
	Growth and development in initial weeks and months of life	x	x	no	x	x	no	x	x	x	x
	Protocols for screening for metabolic conditions, infectious conditions, and congenital abnormalities	no	x	x	x	no	x	x	no	no	x
	Protocols/guidelines for immunizations in infancy	no	no	x	x	x	no	no	x	x	x
	Evidence-based information about infant circumcision; family values, beliefs, and cultural norms	no	no	no	no	no	no	no	x	x	no
Skills and behaviours	Examine infant at frequent intervals to monitor growth and developmental behaviour	no	x	no	x	x	no	x	x	x	x
	Distinguish normal variation in newborn appearance and behaviour from those indicating pathologic conditions	x	x	x	x	no	x	x	x	x	x
	Administer immunizations, carry out screening tests as indicated	no	no	x	x	x	no	no	x	x	x
	Provide information to parents about a safe environemnt for infant, frequent feeding, care of umbilical cord, voiding and stooling, and close physical contact	no	x	x	x	no	x	x	x	x	no
**4c: Promote and support breastfeeding**											
Knowledge	Physiology of lactation	x	x	x	no	no	x	no	x	x	x
	Nutritional needs of newborn infants, including low birth weight infants	x	x	x	no	no	no	x	x	x	x
	Social, pscychological, and cultural aspects of breastfeeding	no	no	no	no	no	no	no	no	x	no
	Evidence about benefits of breastfeeding	no	x	x	no	no	x	x	no	x	no
	Indications and contraindications to use of drugs and substances during lactation	no	no	no	no	no	no	x	no	no	no
	Awareness of lactation aids	no	x	no	no	no	no	no	no	no	no
Skills and behaviours	Promote early and exclusive breastfeeding while respecting a woman's choice regarding newborn feeding	no	x	x	no	no	x	x	x	no	x
	Provide information about infant feeds, frequency and duration of feedings, and weight gain	no	x	x	no	no	no	x	x	no	no
	Provide support and information about breastfeeding for a minimum of six months, including combining with work, maintaining milk supply, and storing breast milk	no	?	no	no	no	no	x	x	no	x
	Identify and manage breastfeeding problems (e.g. mastitis, low milk supply, engorgement, improper latch)	no	x	x	x	x	x	x	x	x	x
	Provide information to women breastfeeding multiple newborns	no	x	no	no	no	no	no	no	no	no
	Refer women to breastfeeding support as indicated	no	x	no	no	no	no	no	no	x	no
	Advocate for breastfeeding in family and community	no	x	no	no	no	no	no	x	no	no
**4d: Detect, treat and stabilise postnatal complications in woman and refer as necessary**											
Knowledge	Signs and symptoms of: - conditions in the postnatal period that may respond to early intervention (e.g. sub-involution, anaemia, urinary retention, and localized infection), - complications that need referral to more specialized provider or facility (e.g. hematoma, thrombophlebitis, sepsis, obsetetric fistula, and incontinence), - life threatening complications requiring immediate response and specialized care (hemorrhage, amniotic fluid embolus, seizure, and stroke)	x	x	x	x	x	x	x	x	x	x
	Signs and symptoms of postnatal depression, anxiety, and psychosis	no	x	no	x	x	no	x	no	x	x
	Mourning process following perinatal death	no	x	x	no	no	no	x	x	x	no
Skills and behaviours	Provide information to woman and family about potential complications and when to seek help	x	x	no	x	x	x	x	x	no	x
	Assess woman during postnatal period to detect signs and symptoms of complications	x	x	x	x	x	x	x	x	x	x
	Distinguish postnatal depression from transient anxiety about caring for baby, assess availability of help and support at home, and provide emotional support	no	no	no	no	no	no	x	no	x	no
	Provide counselling and follow-up care for women and family members who experience stillbirth, neonatal death, serious infant illness and congenital conditions	no	x	x	no	no	no	no	x	no	no
	Provide first line measures to treat or stabilise identified conditions	x	x	x	x	x	x	x	x	x	x
	Arrange referral and/or transfer as needed	x	x	x	x	x	no	x	x	x	x
**4e: Detect, stabilise, and manage health problems in newborn infant and refer if necessary**											
Knowledge	Congenital abnormalities, and genetic conditions	no	x	x	x	no	x	x	x	x	x
	Needs of preterm and low birth weight infants	no	x	x	x	no	no	x	x	no	x
	Symptoms and treatment of withdrawal from maternal drug use	no	no	no	no	no	no	no	x	no	no
	Prevention of mother-to-child transmission of infections such as HIV, hepatitis B and C	x	x	x	x	x	x	x	x	x	x
	Signs and symptoms of common health problems and complications; their immediate and ongoing treatment	x	x	x	x	x	x	x	x	x	x
Skills and behaviours	Assess and recognise abnormal findings	x	x	x	x	x	x	x	x	x	x
	Implement protocols for care of low birth weight infants, e.g. ensure warmth, nutrition, monitor condition, "Kangaroo" care as appropriate	no	x	x	x	x	no	x	x	no	x
	Provide information to mother and support persons about infant condition	no	x	x	x	x	no	x	x	no	no
	Provide support in situations where infant separated from mother for special care	no	x	no	x	no	no	x	x	no	no
	Recognise indications of the need for specialized care in the newborn	no	x	x	x	no	x	x	x	x	x
	Stabilise and transfer the at-risk newborn to emergency care facility	x	x	x	x	no	no	x	x	x	x
**4f: Provide family planning services**											
Knowledge	Anatomy and physiology of female and male related to reproduction and sexual development	x	x	x	x	x	x	x	x	x	x
	Socio-cultural aspects of human sexuality	no	x	no	no	no	x	x	no	x	no
	Family planning methods including natural, barrier, hormonal, implantable, emergency contraception, sterilization; their possible side effects, risk of pregnancy, and contraindications to use	x	x	x	x	x	x	x	x	x	x
	Available written and pictorial resources for teaching about family planning methods	x	no	x	x	x	x	no	x	x	x
	Pregnancy options for HIV positive women or couples	x	x	no	no	no	no	no	no	x	no
Skills and behaviours	Provide and protect privacy and confidentiality for discussions about family planning knowledge, goals for limiting and/or spacing of children, and concerns and myths about methods	no	x	x	no	no	x	x	x	x	x
	Obtain relevant history of use of methods, medical conditions, socio-cultural values, and preferences that influence choice of method	x	x	x	x	x	x	x	x	x	x
	Provide information about how to use, effectiveness, and cost of various methods to support informed decision making	x	x	x	x	x	x	x	x	x	x
	Provide methods according to scope of practice and protocols, or refer to another provider	x	x	x	x	x	x	no	x	x	x
	Provide follow-up assessment of use, satisfaction, and side effects	no	no	no	no	no	no	no	x	no	no
	Refer for woman or partner for sterilization procedure	no	no	no	no	no	no	no	x	no	no
