# Supplementary material for: Are midwives ready to provide quality evidence-based care after pre-service training? Curricula assessment in four countries—Benin, Malawi, Tanzania, and Uganda
Source: PLOS Glob Public Health. 2022 Sep 19;2(9):e0000605. doi: 10.1371/journal.pgph.0000605 (PMC10021168; doi:10.1371/journal.pgph.0000605)
Supplement: Attachment — Submitted filename: Response to reviewers.docx [file pgph.0000605.s005.docx]

**Benin:**

Maquette Pédagogique de l’Offre de Formation de Licence Professionnelle en Sciences Infirmières et Obstétricales Option Sage-Femme. Université d’Abomey-Calavi.

The contact Université d’Abomey-Calavi, email: vrcireip.uac@uac.bj.

**Malawi :**

BSc in Nursing and Midwifery. The Daeyang College of Nursing and Nursing.

The Contact Daeyang College, email: [Registrar@dyuni.ac.mw](mailto:Registrar@dyuni.ac.mw).

Midwifery Technicians (NMTs). The Christian Health Association of Malawi (CHAM).

The contact Pachalo Matchere, email: [pmatchere@cham.org.mw](mailto:pmatchere@cham.org.mw).

**Tanzania:**

On-line:

Level 5. Technician Certificate in Nursing and Midwifery (<https://www.stmagdalenenursingschool.com/wp-content/uploads/2019/04/Curriculum-for-NTA-Level-5-Nursing.pdf>).

Level 6. Ordinary Diploma Nursing and Midwifery (<https://www.stmagdalenenursingschool.com/wp-content/uploads/2019/04/Curr_NTA-Level-6.pdf>).

Revised Competency-based Curriculum for the Bachelor of Science in Nursing (BSCN) Programme. Muhimbili University of Health and Allied Sciences (MUHAS).

Competency - Based Curriculum for Bachelor of Science in Midwifery. Muhimbili University of Health and Allied Sciences (MUHAS).

The contact person: The Vice Chancellor, Muhimbili University of Health and Allied Sciences, email: vc@muhas.ac.tz.

**Uganda:**

BSc of science in midwifery (upgrading) programme. Aga Khan University School of Nursing and Midwifery. The contact person: Grace Edwards, email: [grace.edwards@aku.edu](mailto:grace.edwards@aku.edu).

BSs midwifery. Lira University. Contact: Lira University, email : [vc@lirauni.ac.ug](mailto:vc@lirauni.ac.ug).

Diploma in midwifery. Ministry of Education, Science, Technology and Sports.

Contact: Ministry of Education Science, Technology and Sports, email: [info@unmeb.go.ug](mailto:info@unmeb.go.ug).
